# Dietary Exposure to Toxic Metals (Cd, Pb and Hg) from Cereals Marketed in Madeira and the Azores

**DOI:** 10.1007/s12011-023-03643-x

**Published:** 2023-03-21

**Authors:** Carmen Rubio, Ángel J. Gutiérrez, Arturo Hardisson, Verónica Martín, Consuelo Revert, Paulo Jorge Pestana Fernandes, David João Horta Lopes, Soraya Paz-Montelongo

**Affiliations:** 1 Grupo de Investigación en Toxicología Alimentaria Y Ambiental, Universidad de La Laguna, La Laguna, 38071 Tenerife, Islas Canarias Spain; 2 Laboratorio de Salud Pública de Las Palmas, Servicio Canario de Salud, 35004 Las Palmas de Gran Canaria, Spain; 3 Departamento de Medicina Física Y Farmacología, Universidad de La Laguna, 38071 Tenerife, Islas Canarias Spain; 4 Divisão de Análises de Resíduos E Contaminantes. Secretaria Regional de Agricultura E Desenvolvimento Rural. Direção Regional de Agricultura E Desenvolvimento Rural. Direção de Serviços Dos Laboratórios Agrícolas E Agroalimentares, Laboratório Regional de Veterinária E SegurançaAlimentar. Caminho das Quebradas de Baixo, N.º 79 – 9000-254 Funchal, Madeira Portugal; 5 Ce3C - Centre for Ecology, Evolution and Environmental Changes, Azorean Biodiversity Group, Faculty of Agricultural Sciences and Environment, University of the Azores, Ponta Delgada, Portugal

**Keywords:** *Cereals*, *Toxic Metals*, *Pb*, *Cd*, *Hg*, *Exposure Assessment*, *Risk Characterization*, *Madeira*, *Azores*, *Macaronesia*

## Abstract

Cereals and cereal-based foods continue to be basic foods in all diets. Despite being known for their high nutritional value; they can also contain contaminants (hazards) such as toxic metals. This study assesses the Cd, Pb and Hg dietary exposure from cereals and derivatives marketed in Madeira and the Azores and characterizes the risks by evaluating the Cd and Hg intake contributions to the tolerable intakes and by estimating the Margin of Exposure (MOE) in the case of Pb. In Madeira, metals follow the descending order of Cd > Pb > Hg. Cd stands out as having the highest levels (0.307 mg Cd/kg in oats; 0.237 mg/kg in rye). High levels of Pb (0.347 mg/kg) were also detected in rye. Regarding total mercury, rice stands out (0.0013 mg/kg) followed by wheat (0.001 mg/kg). While all cereals and derivatives except maize consumed in Madeira exceed the maximum value of Cd allowed by the EU, 50.0% of the rye and 25.0% of the corn flour samples exceeded the European Pb limit. The daily consumption of 100 g of oats, rye flour and rye represent high contributions to the TWI of Cd (93.2 – 120%). The MOE values of Pb from the consumption of rye (100 g/day) are 1,294 (nephrotoxic effects) and 3,082 (cardiotoxic effects). In the Azores, corn flour (0.72 mg Pb/kg) stands out with 85.7% of the samples exceeding the maximum Pb EU limit and MOE values of 626 (nephrotoxic effects) and 1,490 (cardiotoxic effects). Regular daily consumption of corn flour makes a low (< 10%) contribution to the Cd TDI. In conclusion, the Pb exposure from the consumption of cereals and derivatives could have toxic effects such as nephrotoxicity or cardiotoxicity in adults. The results highlight the need to set up monitoring and surveillance programs for the safety of cereals and their derivatives in Madeira and the Azores in terms of lead and cadmium.

## Introduction

Agriculture and the cultivation of cereals are the livelihood of an important part of the Portuguese islands population [1] but cereal imports are necessary to supply the local demand in these two Archipelagos. Cereals are a relevant dietary source of not only nutrients but also of pollutants of toxicological interest such as toxic metals (Cd, Pb and Hg) [2, 3]. In 2012, the EFSA classified cereals as one of the food categories that contributes the most to the dietary intake of Cd in the European population [4].

Maximum values ​​have been set to minimize the exposure of European consumers to these food hazards,. The European Regulation (EU) 2021/1323 of the Commission which modified Regulation (EC) 1881/2006, sets a maximum value of Cd of 0.04 mg/kg (in foods made from cereals), 0.05 mg/kg (in rye and barley), 0.15 mg/kg (in rice, quinoa, wheat bran and wheat gluten), 0.18 mg/kg (in Triticum durum; durum wheat) and 0.10 mg/kg (in other cereals) [5]. In the case of Pb, a maximum value of 0.20 mg/kg has been set for cereals and a limit of 0.02 mg/kg for foods made from cereals and baby food for infants and young children according to the European Regulation (EU) 2021/1317 that modified Regulation (EC) 1881/2006 [6]. In the case of Hg, a total mercury limit value of 0.01 mg/kg was established for various cereals (barley, corn, oats, rice, rye, wheat, etc.) by the Commission Regulation 2018/73 which modified annexes II and III of Regulation (EC) 396/2005 regarding the maximum residue limits of mercury compounds in certain products [7].

Human exposure to toxic metals is associated with a wide variety of risks. Cd is a metal that competes with essential elements such as zinc (Zn), copper (Cu) or iron (Fe), and thus it interferes with various metabolic pathways [4, 8]. In addition, Cd is known for its ability to induce metallothionein’s and its high nephrotoxicity [9, 10]. Pb toxicity can be attributed to the affinity of lead for thiol groups (-SH) and other organic ligands in proteins and its ability to substitute Ca and Zn. Inorganic lead compounds have been classified by the International Agency for Research on Cancer (IARC) as probably being carcinogenic to humans (Group 2A) [11]. Although the central nervous system (CNS) is the main target organ for lead toxicity in humans, developmental cardiotoxicity in young children and cardiovascular effects, nephrotoxicity and haematological effects in adults have been identified as being potential critical adverse effects of lead on which to base risk assessment [12–15].

Mercury is a toxic element whose presence in cereals is mainly due to the contamination of soils used for cultivation and to the absorption and accumulation capacity of cereals. Hg is an element whose toxicity depends on its chemical form. Methylmercury is absorbed in the gastrointestinal tract at a rate of about 95% of the total ingested [16]. Chronic exposure to methylmercury via the digestive route is associated with cardiotoxic effects, nephrotoxic effects, damage to the immune and haematological systems, as well as with problems related to embryonic development [16–18].

The minimization of European dietary exposure to these toxic metals has also led the EFSA (European Food Safety Authority) to set tolerable weekly intakes (TWI) for Cd (2.5 µg Cd/kg bw/week) [19] and Hg (4 µg/kg bw /week) [20] and benchmarks for Pb (0.63 µg/kg bw/day for cardiotoxic effects and 1.5 µg/kg bw/day for cardiotoxic effects) [12].

Dietary exposure to lead in twelve EU Member States was estimated to be, on average, 42 μg/day (0.7 μg/kg b.w. per day assuming 60 kg b.w.) in adults [21]. In the Macaronesia region, Rubio et al. [22] evaluated the dietary exposure to Cd in the Canary Islands archipelago, determining the Cd intake from cereals at 1.065 µg/day. Subsequently, Rubio et al. [23] reported data on Cd concentration in cereals marketed in Cape Verde, highlighting the content of this metal in wheat flour (0.02 mg/kg) which generated an estimated daily dietary intake of 2.00 µg/day.

In 2012, lifetime lead exposure in the European population was estimated at 0.68 µg/kg bw/day [18] and cereals were identified as Pb dietary sources. Pb contamination in cereals and derivatives consumed in the Canary Islands and Cape Verde Macaronesia archipelagos has been previously assessed [24, 25]. Pb concentrations in cereals and derivatives consumed in Cape Verde ranged from 0.03 – 0.08 mg/kg and Pb dietary intakes from this food group were estimated to range from 3 – 8 µg/day [26].

Considering the importance of cereals and their derivatives in the human diet, the potential contamination of this food group with toxic metals and the potential health risks derived from the dietary exposure, the aims of this study were to determine the concentration of Cd, Pb and Hg in cereals and derivatives consumed by the populations of the Portuguese Archipelagos of Madeira and Azores, to assess the dietary intakes of these toxic metals from the consumption of cereals and derivatives and to characterize the potential health risks.

## Material and Methods

### Samples

Sixty samples of cereals and derivatives marketed in Madeira (rice, corn flour, rye flour, wheat flour, rye, wheat, oats, corn and couscous) and forty samples of cereals and derivatives marketed in the Azores (corn flour, wheat flour) were collected and analysed (Table 1). The origin of the samples was from EU and non-EU countries. All samples are part of the sampling of the PERVEMAC II project (INTERREG V-A Cooperation Program Spain-Portugal MAC (Madeira-Azores-Canarias) 2014–2020 grant number MAC/1.1a/049).

**Table 1:** Samples of cereals and derivatives marketed in Madeira and the Azores

Type of Cereal or cereal derivative	Number of samples	Origin
Rice	18	Madeira
Oat	11	Madeira
Rye	2	Madeira
Couscous	2	Madeira
Rye flour	4	Madeira
Corn flour	8	Madeira
Corn flour	16	Azores
Wheat flour	8	Madeira
Wheat flour	24	Azores
Corn	3	Madeira
Wheat	4	Madeira
Total samples	100	

### Sampling Area

The Portuguese Autonomous Region of Madeira with an area of 801 km2 and 267,785 inhabitants is made up of two inhabited islands (Madeira and Porto Santo) and three smaller uninhabited islands (Desert Islands).

The Portuguese Azores Archipelago has three group of islands with a total surface area of 600 km2: the Eastern group with two islands: São Miguel and Santa Maria; the Central group, with five islands (Terceira, S. Jorge, Graciosa, Faial and Pico) and the Western group with two islands: Flores and Corvo. Both of these two Atlantic Portuguese archipelagos are considered part of the Macaronesia region.

Samples were obtained from markets on Madeira Island (32°39′00″N 16°55′00″W) and Terceira Island (Azores) (38°44′00″N 27°19′00″W). The climate in Madeira is subtropical, while on the island of Terceira it is an island climate. Both islands are of volcanic origin.

### Sample Treatment

One gram of each homogenized sample was weighed in Teflon tubes (GO for Smart Vent, Anton Parr, Austria) using an analytical precision balance (Mettler Toledo, Spain). Two mL of 30% hydrogen peroxide (H_2_O_2_) (Sigma Aldrich, Darmstadt, Germany) and 4 mL of 65% concentrated nitric acid (HNO_3_) (Sigma Aldrich, Darmstadt, Germany) were added. Samples were subjected to wet digestion in acid medium with microwave digestion system (Multiwave GO, Anton Paar GO, Austria). Three replicates were prepared for each sample. The microwave digestion process is based on a one-hour program divided into stages: i) initial stage of fifteen minutes until reaching 100ºC, maintained for five minutes; ii) second stage of ten minutes until reaching 180 °C, maintained for 15; iii) third stage of fifteen minutes of lowering the temperature to ambient temperature. Finally, the digested samples are placed in 10 ml volumetric flasks and made up to volume with distilled water obtained from the distillation system (Millipore, Burlington, MA, USA) [25].

### Determination of Toxic Metals

The determination of Cd and Pb was performed with an atomic absorption spectrophotometer (AS-800, Perkin Elmer, USA) with a graphite chamber (HGA-800, Perkin Elmer, USA) (GF-AAS). Atomic absorption spectrophotometry (AAS) is the analytical method approved by Regulation 333/2007 (CE) modified by Regulation 836/2011 [27] for the official control of levels of lead, cadmium and mercury in food products.

The determination of Hg was carried out with a cold vapour atomic absorption spectrophotometer (AS-800, Perkin Elmer, USA) (CV-AAS) with a flow injection system (FIMS-400, Perkin Elmer, USA). The instrumental wavelengths (nm) were: Cd (228.8), Pb (283.3), and Hg (253.7); the instrumental limits of quantification (LOQ) of the method were: Cd (0.013 mg/kg), Pb (0.040 mg/kg), and Hg (0.10 mg/kg). Instrumental conditions are shown in Table 2.

**Table 2:** Instrumental conditions of the graphite chamber for Cd and Pb determination and of the cold vapour for Hg determination

Cd	Step	Temp. (ºC)	Ramp time (min)	Hold time (min)	Internal Flow	Gas type
	1	110	10	20	250	Normal
	2	130	15	30	250	
	3	700	10	20	250	
	4	1500	0	5	0	
	5	2450	1	3	250	
Pb	Step	Temp. (ºC)	Ramp time (min)	Hold time (min)	Internal Flow	Gas type
	1	110	1	30	250	Normal
	2	130	15	30	250	
	3	700	10	20	250	
	4	1500	0	5	0	
	5	2450	1	3	250	
	Read step: 4	Injection temp. (ºC): 20				
	Sample (Cd & Pb)					
	Volume: 20 µL		Diluent volume: 0 µL		Diluent location: 131	
Hg	Time (sec)	Pump 1 speed	Pump 2 speed		Valve position	
	15	100	120		Fill	
	11	100	120			
	15	0	120		Inject	

Reference material (NIST SRM 1577 BL, Sigma Aldrich, Germany) was used for the determination of Hg. For Cd and Pb, the reference material NIST1567B Wheat flour (Sigma Aldrich, Germany) was used. The recovery study, subjecting the reference material to the same conditions as the samples, yielded recovery values above 97% in all cases.

### Dietary Exposure Assessment and Risk Characterization

The EDIs (estimated daily intakes) were calculated considering a 100 g/day consumption scenario of the cereal or cereal-based food and the detected concentration of the toxic metal (Eq. 1).

For the Cd and Hg risk characterization, the EFSA Tolerable Weekly Intakes (TWI) were used (Cd: 2.5 µg/kg body weight/week [19]; Hg: 4 µg/kg body weight/week [20]. Equation 2 was used to estimate the contribution percentage of the estimated intakes to the TWI.

The risks to human health related to the presence of Pb in cereals and derivatives were characterized by applying the Margin of Exposure (MOE) since the EFSA CONTAM Panel concluded in 2010 that the provisional tolerable weekly intake (PTWI) of 25 μg/kg b.w. was no longer appropriate as there was no evidence for a threshold for several critical endpoints including developmental cardiotoxicity and nephrotoxicity in adults. The BMDL values for Pb nephrotoxicity (0.63 µg/kg body weight/day) and Pb cardiotoxicity (1.50 µg/kg body weight/day) [12] were used as reference values to calculate the MOE (Eq. 3). As shown, the MOE is obtained by dividing the BMDL intake value by the estimated daily intake (EDI).

According to Costa et al. [28], the mean body weight for an adult in the Madeira archipelago is 71.5 ± 15 kg. For the Azores population, there is no specific survey that refers to a body weight. The same body weight (71.5 kg) was considered for the risk characterization to facilitate the discussion and comparison of the results between both archipelagos.

1 $$EDI=\frac{Metal\, content(\frac{mg}{kg})}{Mean\, cereal\, consumption(\frac{kg}{day})}$$

2 $$Contribution \left(\mathrm{\%}\right)=\frac{\text{EDI}}{{\text{TWI}}}\cdot 100$$

3 $$MOE=\frac{\text{BMDL}}{{\text{EDI}}}$$

### Statistical Analysis

The statistical program GraphPad Prism 8.4.3 (GraphPad Prism, San Diego, CA, USA) for Windows™ was used to detect possible significant differences in the content of toxic metals between the cereals and their derivatives. The Anderson–Darling, D'Agostino and Pearson, Shapiro–Wilk and Kolmogorov–Smirnov tests were applied to study the normality of the data [29–31]. Given that the data did not follow a normal distribution, the non-parametric Mann–Whitney test was applied, and p < 0.05 values were considered statistically significant.

## Results and Discussion

### Madeira: Metal Contents, Exposure Assessment and Risk Characterization of Cereals and Derivatives

Table 3 shows the mean average concentration of toxic metals (Cd, Pb, Hg) in the different cereals and derivatives marketed in Madeira. In general, the toxic metals analysed follow the descending order of Cd > Pb > Hg. As shown in Table 3, corn is the only cereal that does not exceed the EU value for Cd, and rice, oat, wheat flour, rye flour, wheat, corn, couscous do not exceed the EU limit value for Pb.

**Table 3:** Madeira: mean concentrations (mg/kg) and standard deviations (SD) by type of cereal and derivative

Cereal/Cereal-based product	No. samples	Cd (mg/kg)	EU Cd max. Levels exceeded^a^	Samples over the Cd limits (%)	Pb (mg/kg)	EU Pb max. levels exceeded	Samples over the EU Pb limits (%)	Hg (mg/kg)	EU Hg max. levels exceeded ^c^
Rice	18	0.164 ± 0.02	Yes	44.4	0.023 ± 0.002	No	0	0.0013 ± 0.0002	No
Oat	11	0.307 ± 0.03	Yes	72.7	0.012 ± 0.001	No	0	0.00022 ± 0.00002	No
Corn flour	8	0.147 ± 0.02	Yes	50.0	0.019 ± 0.002	Yes	25.0	0.00012 ± 0.00001	No
Wheat flour	8	0.222 ± 0.02	Yes	62.5	0.0021 ± 0.0002	No	0	0.00009 ± 0.000005	No
Rye flour	4	0.275 ± 0.02	Yes	75.0	0.0075 ± 0.0006	No	0	0.0002 ± 0.00002	No
Wheat	4	0.119 ± 0.01	Yes	25.0	0.0133 ± 0.001	No	0	0.001 ± 0.00007	No
Corn	3	0.029 ± 0.003	No	0	0.0126 ± 0.002	No	0	0.0001 ± 0.00001	No
Rye	2	0.238 ± 0.02	Yes	100	0.3475 ± 0.04	Yes	50.0	0.0001 ± 0.00001	No
Cous-cous	2	0.0425 ± 0.005	Yes	50.0	0.014 ± 0.002	No	0	0.0001 ± 0.00001	No

^a^Maximum levels of Cd: 0.04 mg/kg (cereal-based foods), 0.05 mg/kg (rye and barley), 0.15 mg/kg (rice, quinoa, wheat bran and wheat gluten), 0.18 mg/kg (Triticum durum; durum wheat) y 0.10 mg/kg (rest of cereals) [5]. ^b^Maximum levels of Pb: 0.20 mg/kg (cereals, pulses and dried pulses), 0.02 mg/kg (processed cereal-based foods and baby foods for infants and young children) [6]. ^c^Maximum levels of total Hg: 0.01 mg/kg (barley, maize, oats, rice, rye, wheat, etc.) [7]

In Madeira, Cd stands out for presenting the highest levels in all the cereals and derivatives registering the highest mean average concentration in oats (0.307 mg Cd/Kg) and rye (0.237 mg/kg). The statistical study shows the existence of significant differences (p < 0.05) in the Cd content between rice vs. oats (p = 0.0479). Considering the maximum values ​​allowed in the European regulations [5, 6], almost all the samples analysed exceed the maximum value of Cd. Only maize is below the maximum legal limit. Similar results were observed in Burkina Faso by Bazie et al. [32] where Cd was detected in 92.5% of rice samples and in 44.44% of maize samples and more than half of the samples showed Cd concentrations above the limits set by the Codex Alimentarius. The abovementioned authors reported that the population of Burkina Faso is exposed to a non-cancer risk linked to metallic trace elements associated with rice, maize and peanut consumption [32]. The results of the present study are similar to those observed in Poland where Cd was found to have values of 0.27 ± 0.03; 0.2 ± 0.01; 0.31 ± 0.01 mg Cd/kg for maize, barley, and wheat, respectively [33]. Previous studies conducted by Rubio et al. [23] in the Macaronesia Archipelagos of Cape Verde (2021) detected lower ranges of Cd concentrations for cereals (0.003 – 0.0019 for Cape Verde). Other studies by Rubio-Armendáriz et al. [25], in which Cd was determined in cereals from the Canary Islands, concentrations of 0.040 mg/kg were found in wheat and 0.001 mg/kg in corn. Lower levels of Cd in wheat grain (mg Cd/kg fresh weight) have also been reported in UK (0.038), France (0.045–0.058), The Netherlands (0.060), Sweden (0.049–0.060), Germany (0.056) [34, 35]. Cd levels in Nigeria [36] were also lower in maize (0.006 mg/kg), rice (0.011 mg/kg), and millet (0.008 mg/kg). The same was observed for other Sub-Sahara regions [37] in maize (0.0004 mg/kg) and rice (0.004 mg/kg). In Peru, Roman-Ochoa et al. [38] studied the levels of Cd in several metals and derivatives and the total mean average Cd levels (mg/kg) in grains, maize and rice were also much lower: 0.03 ± 0.02; 0.00 ± 0.01 and 0.11 ± 0.06, respectively.

Regarding lead contamination in cereals and derivatives in Madeira, rye had the highest Pb levels (0.347 mg/Kg). Significant differences (p < 0.05) were recorded for Pb between rice vs. wheat flour (p = 0.0086), rye vs. wheat flour (p = 0.0222), wheat vs. corn flour (p = 0.0406) and wheat vs wheat flour (p = 0.0242). Considering the current EU legislation regulating the content of toxic metals in food for human consumption [5–7], 50.0% of the rye samples and 25.0% of the corn flour samples exceeded the Pb limit set for cereals (0.20 mg/kg). This hazard was also recently observed in Burkina Faso where lead was found in 82.5% of rice samples and 72.22% of maize samples and more than half of the samples had Pb concentrations above the limits set by the Codex Alimentarius [32]. In Poland, Baranowska et al. [33] analysed Pb in several cereals observing even higher levels (4.56 ± 0.12; 1.56 ± 0.52; 1.05 ± 0.3 mg Pb/kg for maize, barley and wheat, respectively). Previous studies in the Macaronesia region reported higher concentrations of Pb in derivatives such as barley gofio (0.520 mg/kg) marketed in the Canary Islands [25]. Cereals and derivatives marketed in Cape Verde [23] also showed slightly higher Pb concentrations (0.03 – 0.08 mg/kg) than those recorded in Madeira, except for rye. On the other hand, maize (0.046 mg/kg) consumed in Nigeria [36], maize (0.007 mg/kg) and rice (0.004 mg/kg) consumed in different Sub-Sahara regions [37] and grains, maize, maize derivatives and rice (0.03 ± 0.01; 0.01 ± 0.01; 0.75 ± 0.60 and 0.04 ± 0.03 mg/kg of Pb, respectively) consumed in Perú [38] presented lower Pb values than the ones detected in Madeira in the present study.

Regarding total mercury, rice stands out for its Hg content (0.0013 mg/kg) followed by wheat (0.001 mg/kg). In China, studies conducted by Zhao et al. [39] report higher Hg values (brown rice: 0.042 mg/kg) than those observed in the present study. Statistical differences (p < 0.05) in Hg content were found here between rice vs oats (p = 0.0043), rice vs corn flour (p = 0.0043), rice vs wheat flour (p = 0.0039).The EU legislation limit (0.01 mg/kg of total mercury in rice) was not exceeded by any of the samples [7].

The differences found are due to multiple factors such as the origin of the samples, the intrinsic characteristics of the plants (uptake, accumulation and metabolism), the presence of these elements in the crop soil and in the irrigation water [2, 3, 23, 40–42]. Whereas, in the case of cereal derivatives, the differences found, in addition to the factors mentioned above, may be due to the addition of other ingredients (additives, salt, anti-caking agents, etc.) as well as to the processes to which they are subjected to obtain the derivative, such as roasting, dehulling, milling, among others.

**Fig. 1:**
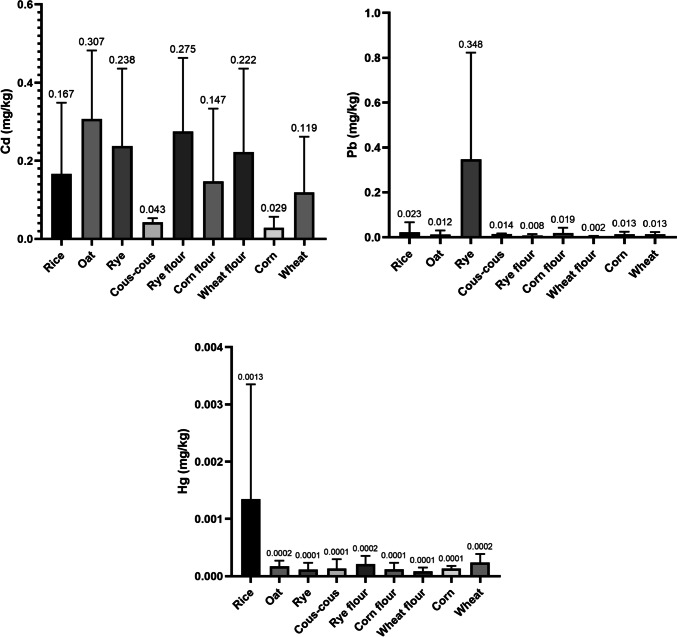
Comparison of the content of toxic metals (Cd, Pb and Hg) between cereals and derivatives marketed in Madeira

Figure 1 shows the comparison in the content of toxic metals (Cd, Pb and Hg) between the different cereals and derivatives.

The Cd, Pb and Hg dietary exposure assessment from cereals and derivatives marketed in Madeira are shown in Table 4. The estimated daily intakes (EDIs) were assessed considering a 100 g/day consumption. In the case of Cd, the consumption of 100 g/day of oats, rye flour and rye represent contributions close to the total TWI (2.5 µg Cd/kg bw/week) with some percentages of contribution ranging from 93.2 to 120.2% for an adult of 71.5 kg of body weight. In the case of Hg, the consumption of 100 g/day represents a contribution to the TWI of less than 1% in all cases and as such does not pose any health risk for adults.

**Table 4:** Cd, Pb and Hg exposure assessment and risk characterization of cereals and derivatives in Madeira in a 100 g/day consumption scenario

Cereal or derivative	Cd		Pb			Hg	
	EDI (100 g/day)	TWI (%)	EDI (100 g/day)	MOE *Nephrotoxicity*	MOE *Cardiotoxicity*	EDI (100 g/day)	TWI (%)
Rice	0.0164	64.2	0.00230	19,585	46,630	0.000130	0.045
Oats	0.0307	120.2	0.00120	37,538	89,375	0.000020	0.007
Corn flour	0.0147	57.6	0.00190	23,708	56,447	0.000010	0.003
Wheat flour	0.0222	86.9	0.00020	225,225	536,250	0.000010	0.003
Rye flour	0.0275	107.7	0.00080	56,306	134,063	0.000020	0.007
Wheat	0.0119	46.6	0.00130	34,650	82,500	0.000100	0.035
Corn	0.0029	11.4	0.00130	34,650	82,500	0.000010	0.003
Rye	0.0238	93.2	0.03480	1294	3082	0.000010	0.003
Couscous	0.0043	16.8	0.00140	32,175	76,607	0.000010	0.003

^a^Maximum tolerable values: Cd (TWI: 2.5 µg/kg body weight/week) [19]; Pb (BMDL nephrotoxicity: 0.63 µg/kg body weight/day; BMDL cardiotoxicity: 1.50 µg/kg body weight/day) [12]; Hg (TWI: 4 µg/kg body weight/week) [20].

^b^EDI, estimated daily intake; TWI, tolerable weekly intake; BMDL, benchmark dose level.

^c^Mean average adult weight: 71.5 kg

In the case of Pb, for the risk characterization, the authors consider that the magnitude of the MOE only indicates a level of concern and does not quantify risk. An MOE value of 10,000 or higher is considered of low concern from a public health point of view with respect to the carcinogenic effect. Therefore, a small MOE represents a higher risk than a larger MOE. In the case of rye, considering the BMDL of 0.63 µg/kg body weight/day, an MOE value of 1294 was obtained, which could indicate potential nephrotoxicity.

### Azores: Metal Contents, Exposure Assessment and Risk Characterization of Cereals and Derivatives

**Fig. 2:**
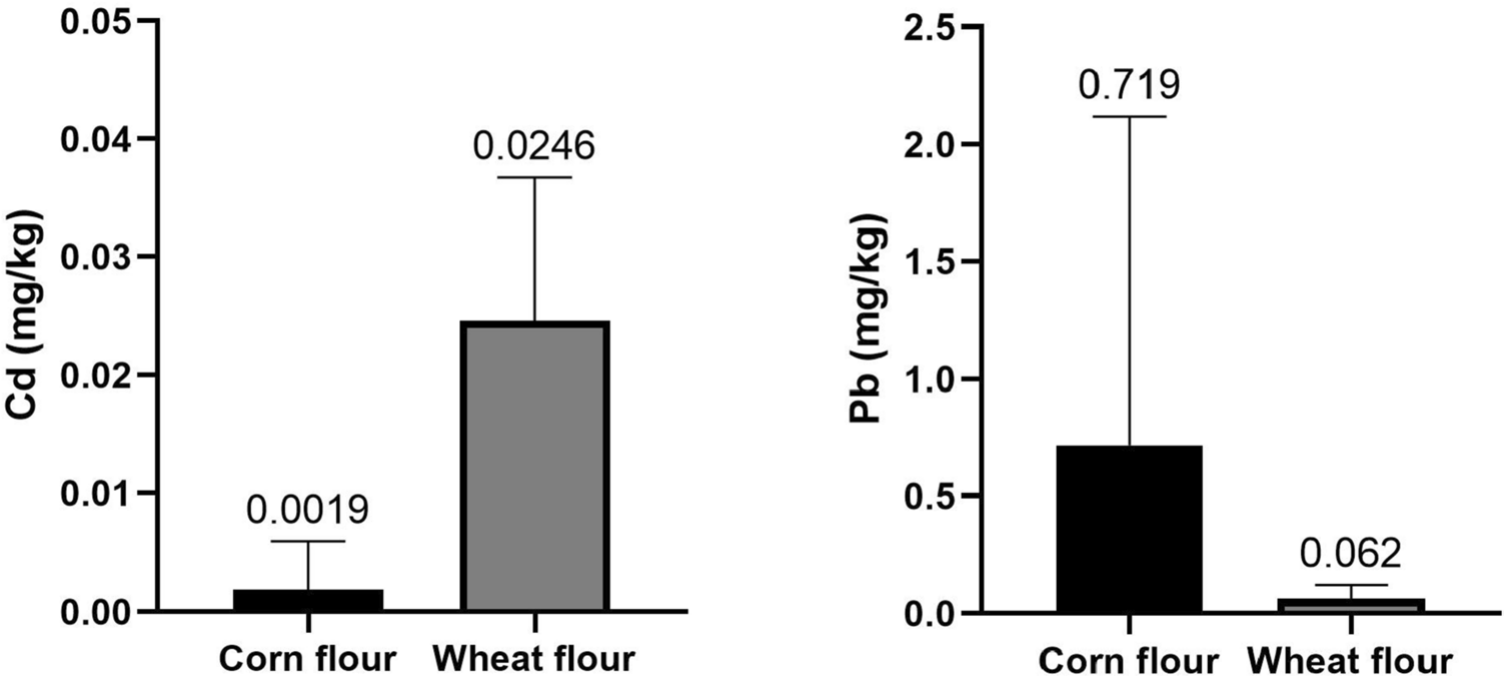
Comparison of the content of toxic metals (Cd and Pb) among the derivatives analysed from the Azores

Table 5 shows the mean average concentrations of Cd, Pb and Hg detected in the cereal’s derivatives sampled in the Azores archipelago. Figure 2 shows the mean Cd and Pb concentrations in corn flour and wheat flours. Wheat flour stands out for its mean Cd content (0.0246 mg/kg) but none of the wheat samples exceeded the EU maximum Cd content [5]. In the case of corn flour, 5.3% of the analysed samples exceeded the EU limit (0.04 mg/kg of Cd) [5]. The statistical study confirmed the existence of significant differences (p < 0.05) in the Cd content between corn flour vs. wheat flour (p = 0.0005) (Fig. 2). The mean Pb content found in corn flour was 0.719 mg/kg and 85.7% of the samples exceeded the EU maximum limit (0.02 mg/kg) [6] and no significant differences were recorded between corn flour vs. wheat flour. Hg contents were all below the limit of detection (LOD).

**Table 5:** Mean concentrations (mg/kg) and standard deviations (SD) in derivatives from the Azores

Cereal or derivative	Cd (mg/kg)	EU Cd max. level exceeded ^a^	Samples over the EU Cd limit (%)	Pb (mg/kg)	EU Pb max. level exceeded^b^	Samples over the EU Pb limit (%)	Hg (mg/kg)
Corn flour	0.0188 ± 0.004	Yes	5.3	0.719 ± 1.398	Yes	21.0	< LOD
Wheat flour	0.0246 ± 0.012	No	0	0.062 ± 0.0578	Yes	85.7	< LOD

^a^Maximum levels of Cd: 0.04 mg/kg (processed cereal-based foods), 0.05 mg/kg (rye and barley), 0.15 mg/kg (rice, quinoa, wheat bran and wheat gluten), 0.18 mg/kg (Triticum durum; durum wheat) and 0.10 mg/kg (other cereals) [5]. ^b^Maximum levels of Pb: 0.20 mg/kg (cereals, pulses and dried pulses), 0.02 mg/kg (processed cereal-based foods and baby foods for infants and young children) [6]

The Cd and Pb exposure assessment for the Azores archipelago is shown in Table 5. As mentioned in the Methodology section, a daily consumption of 100 g and a 71.5 kg body weight were considered. Regarding Cd, the consumption of 100 g/day of corn flour does not imply significant dietary contributions to the TDI of Cd at 2.5 µg/kg bw/week since this is lower than 10%. However, in the case of Pb, the consumption of 100 g/day of corn flour could pose a health risk, considering the BMDL of Pb for nephrotoxic effects, because the MOE value is 626 Table 6.

**Table 6:** Cadmium dietary intake assessment from cereals and derivatives consumed in the Azores

Cereal or derivative	Cd		Pb		
	EDI (100 g/day)	TWI (%)	EDI (100 g/day)	MOE *Nephrotoxicity*	MOE *Cardiotoxicity*
Corn flour	0.00188	7.36	0.072	626	1490
Wheat flour	0.00246	9.63	0.006	7508	17,875

EDI, estimated daily intake; TWI, tolerable weekly intake; BMDL, benchmark dose level

The differences in the levels of metals in the cereals consumed between the different Macaronesia regions show that cereals are imported from different production areas and that each Macaronesia region should follow individual risk management strategies but they should be coordinated.

### Comparison Between Madeira and Azores

**Fig. 3:**
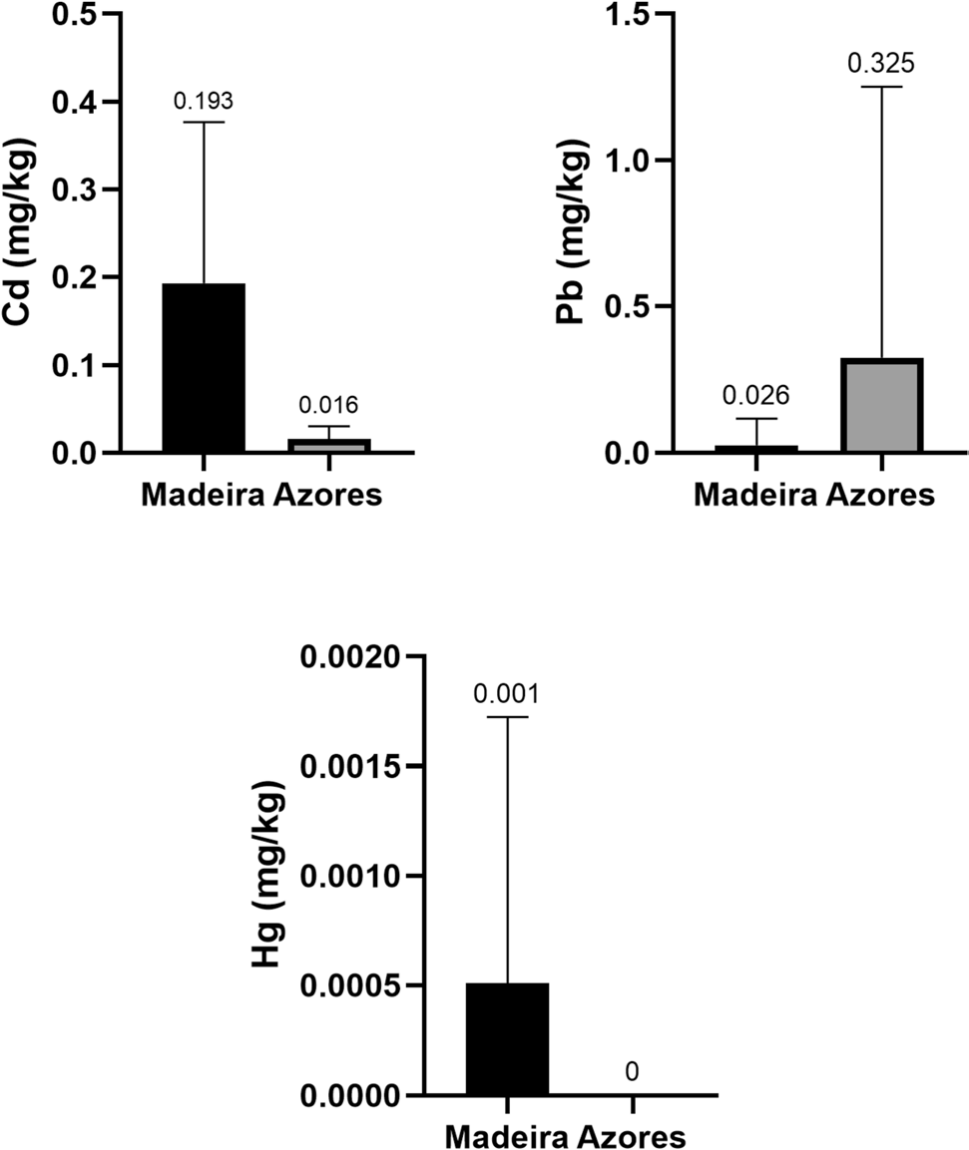
Comparison of Cd, Pb and Hg content in Madeiran and Azorean cereals

The content of toxic metals has been compared between Madeira and Azores cereals, in order to determine the existence of significant differences. Figure 3 shows the comparison between the two regions (Madeira and Azores) in terms of toxic metal content (Cd, Pb, Hg).

In terms of Cd content, the average content recorded in the Madeira region (0.193 mg/kg) stands out in comparison with that detected in cereals and derivatives from the Azores (0.016 mg/kg). While, in the case of Pb, the content of this metal in the products collected in the Azores stands out (0.325 mg/kg). Finally, mercury is only detected in the Madeira samples, with an average concentration of 0.001 mg/kg.

The statistical study has shown the existence of significant differences in the content of the three toxic metals (Cd, Pb, Hg) between Madeira and Azores cereals and derivatives, with a p-value < 0.001.

The differences between cereals in the two regions may be due to multiple factors. For example, in the case of Cd, whose highest concentration was found in Madeira, this may be due to the fact that the samples from this region included cereals such as rice or oats, which tend to accumulate higher concentrations of this metal [42]. In the case of Pb, it is found that the content in the products collected in the Azores stands out, this may be due to the fact that the samples from this region are wheat and maize flour, which tend to accumulate higher concentrations of this metal [41]. Finally, in the case of mercury, it was only recorded in Madeira products, which may be due to the fact that the set of samples contains products that stand out for high concentrations of this metal, such as rice, among others [40].

## Conclusions

The results obtained show that many samples marketed in Madeira and the Azores exceed the legal limits for toxic metal content (Cd and Pb) established by the EC Regulation No.1881/2006.

Monitoring of these contaminants should be applied to both imported and locally produced cereals and derivatives. Finally, and with the aim of facilitating the risk assessments of these and other food contaminants, the authors recommend updating the food consumption data of the different populations. Therefore, authorities are encouraged to initiate and implement or update studies or surveys of dietary habits of the population of these archipelagos because they may not be similar to the continental Portuguese populations.
